# Preoperative Endoscopic Activity Predicts the Occurrence of Pouchitis After Ileal Pouch–Anal Anastomosis in Ulcerative Colitis: A Multicenter Retrospective Study in China

**DOI:** 10.3389/fsurg.2021.740349

**Published:** 2021-09-23

**Authors:** Weimin Xu, Wenbo Tang, Wenjun Ding, Hang Hu, Wenhao Chen, Qun Qian, Long Cui, Zhao Ding, Peng Du

**Affiliations:** ^1^Department of Colorectal Surgery, School of Medicine, Xinhua Hospital, Shanghai Jiaotong University, Shanghai, China; ^2^Department of Colorectal and Anal Surgery, Hubei Key Laboratory of Intestinal and Colorectal Diseases, Zhongnan Hospital of Wuhan University, Wuhan, China

**Keywords:** ulcerative colitis, pouchitis, ulcerative colitis endoscopic index of severity, mayo endoscopic score, IPAA

## Abstract

**Background:** Pouchitis is the most common long-term complication after ileal pouch–anal anastomosis (IPAA) in patients with ulcerative colitis (UC). Ulcerative colitis endoscopic index of severity (UCEIS) and Mayo endoscopic score (MES) are widely used indices to evaluate endoscopic activity. This study aimed to clarify the predictive value of preoperative endoscopic activity on the occurrence of pouchitis after IPAA.

**Methods:** Data of patients with UC who underwent IPAA from January 2008 to January 2020 were collected retrospectively. UCEIS and MES were based on the preoperative colonoscopy findings of two independent endoscopists.

**Results:** A total of 102 patients with a median follow-up of 5 (interquartile range, 2–9) years were included in the study. Among them, 21.6% developed pouchitis. Compared with MES, UCEIS had a stronger correlation with pouchitis disease activity index. UCEIS ≥ 7 had the most significant receiver-operating characteristic (ROC) curve area of 0.747 with a sensitivity of 68.2% and specificity of 81.2% in predicting pouchitis, which outperformed MES of 3 with an ROC area of 0.679 with a sensitivity of 54.5% and specificity of 81.2%. Furthermore, we found that UCEIS ≥ 7 was an independent risk factor for post-IPAA pouchitis [odds ratio (OR), 8.860; 95% CI, 1.969–39.865, *p* < 0.001] with a higher risk than MES of 3 (OR, 5.200; 95% CI, 1.895–14.273; *p* = 0.001).

**Conclusion:** Ulcerative colitis endoscopic index of severity performed better in predicting pouchitis after IPAA than MES. Earlier and more frequent postoperative colonoscopic surveillance should be considered in patients with preoperative UCEIS ≥ 7 to detect the occurrence of pouchitis earlier.

## Introduction

Ulcerative colitis (UC) is a chronic intestinal inflammatory disease, which is characterized by recurrent relapse and remission (1). Total proctocolectomy with ileal pouch–anal anastomosis (IPAA), first proposed in 1978 (2), involved the construction of a “J” pouch to maintain the continuity of the intestine and prevent permanent ileostomy. IPAA was considered as a radical surgery for patients with UC (2).

Although IPAA can significantly improve the long-term quality of life (3–5), the postoperative complications are inevitable. Pouchitis is the most common late complication of IPAA (6, 7), which seriously compromises the prognosis of the patients. Moreover, the etiology of pouchitis remains unclear (8). The pouchitis disease activity index (PDAI), revised in 1994 (9), is widely used in clinical practice to assess the clinical manifestation, endoscopic examination, and histologic findings of the pouch (10). Exploring appropriate indicators to predict the occurrence of post-IPAA pouchitis has become increasingly important.

Preoperative colonoscopy is necessary for each patient to evaluate endoscopic activity. However, data on the predictive value of the two most commonly used indices, namely, ulcerative colitis endoscopic index of severity (UCEIS) and the Mayo endoscopic score (MES) are scarce (11–13). Thus, we aimed to clarify the predictive value of preoperative endoscopic activity on the occurrence of pouchitis after IPAA. We performed this study to discover the incidence of post-IPAA pouchitis in our institute. Then, we compared the predictive values of UCEIS and MES for pouchitis to establish the best cut-off indicators for early colonoscopic surveillance after IPAA to predict the occurrence of pouchitis.

## Methods

### Patients

Some consecutive patients with UC who received long-term and regular medical treatment from January 2008 to January 2020 at our inflammatory bowel disease (IBD) surgery centers (Department of Colorectal Surgery, Xinhua Hospital, Shanghai Jiaotong University School of Medicine and Department of Colorectal and Anal Surgery, Zhongnan Hospital of Wuhan University) and IBD-medicine treatment center (Department of Gastroenterology and Rui-Jin Hospital, Shanghai Jiao Tong University School of Medicine) were considered to participate in this study. All eligible patients with UC who underwent IPAA in our IBD surgery centers or had regular follow-up after IPAA in our IBD medicine treatment center were ultimately enrolled in this study.

The baseline characteristics of patients, medical history, examination data, surgical information, preoperative endoscopic activity, and post-IPAA complications were retrospectively collected from medical records of hospital, outpatient examination, long-term regular follow-up, and the prospectively maintained, institutional review board-approved pouch database (3).

### Inclusion and Exclusion Criteria

Patients older than 18 years who had received standard IPAA with pouch construction, had regular follow-up, and had complete clinical data were included in the study.

Patients who developed complications following symptomatic surgery such as subtotal colectomy with temporary or permanent ileostomy without pouch construction, those diagnosed with Crohn's disease and familial adenomatous polyposis, and those lost to follow-up or with incomplete clinical data were excluded.

### UCEIS and MES Evaluation

In our institution, hospitalized patients with UC routinely undergo a colonoscopy before surgery. The UCEIS and MES were recorded by two independent and experienced endoscopists who received formal and appropriate training to evaluate endoscopic activity. When the endoscopic activity of the same affected area was inconsistent, the higher value was used in the subsequent analysis. All UCEIS and MES records were collected form the pre-IPAA colonoscopy. As reported in the previous studies, the UCEIS comprises three scoring criteria, namely, vascular pattern (0–2), bleeding (0–3), and erosions and ulcers (0–3) (13). Similarly, MES ranges from 0–3 based on the endoscopic findings (14).

### Clinical Evaluation

The primary outcome was the occurrence of pouchitis after IPAA. The diagnosis post-IPAA was evaluated based on a PDAI score ≥7. UC and UC-associated malignant transformation were dependent on the final pathological results. The extent of UC was also divided into proctitis (E1), left-sided colitis (E2), and pancolitis (E3) according to the Montreal classification system (15). The use of mesalamine, biologics, steroids, and immunomodulators and the levels of hemoglobin (Hb) and albumin (Alb) were recorded preoperatively. In this study, early and late post-IPAA complications were defined by the cut-off of 1 month after pouch surgery.

### Statistical Analysis

SPSS version 19.0 software (IBM 2010, Chicago, Illinois, USA) and GraphPad Prism 5 Software (San Diego, California, USA) were used for statistical analysis and creation of figures. Univariate analyses using the Chi-squared, Fisher's exact and Wilcoxon's rank-sum tests were performed for different variables. Multivariate logistic regression to determine the independent risk factor for post-IPAA pouchitis was performed. Pearson's correlation test was used to explore the relationship among UCEIS, MES, and PDAI. Receiver-operating characteristic (ROC) curve analysis and Kaplan–Meier method with the log-rank test were also performed to analyze the predictive value and pouchitis-free overall rate in UCEIS and MES, respectively. In this study, we considered a *p* < 0.05 significant with two-sided test and CI set at 95%.

### Ethical Considerations

The Ethics Committee has reviewed the study protocol and process as well as the application form. We have certified that this study did not raise any issues of the risk of patients. Moreover, the study was conducted in accordance with the Declaration of Helsinki and was free of ethical problems. The Ethics Committee of Xinhua Hospital approved this study (Approval No. XHEC-D-2020-107).

## Results

### Main Characteristics of the Patients

**Figure 1:**
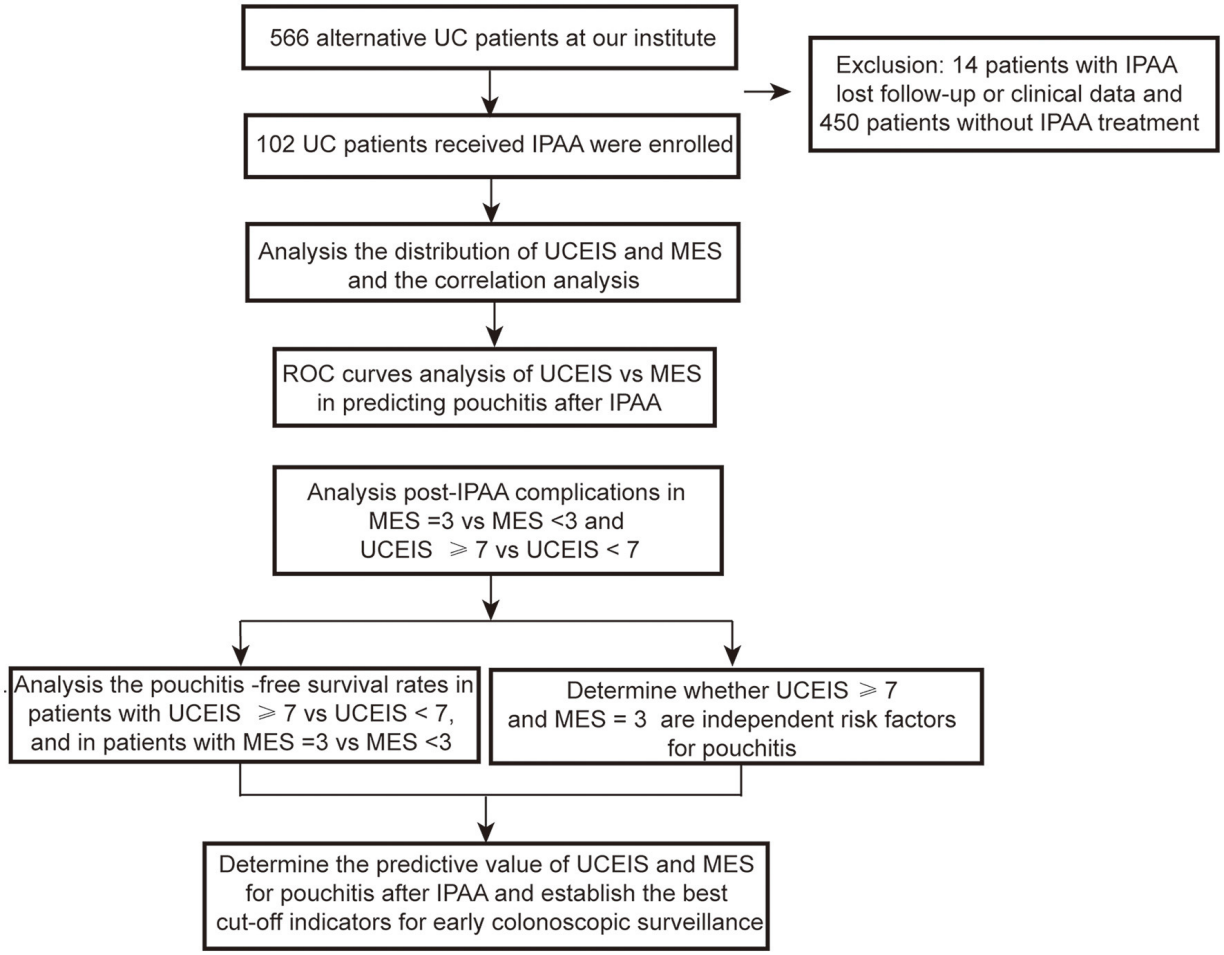
A schematic flow diagram of this study.

**Figure 2:**
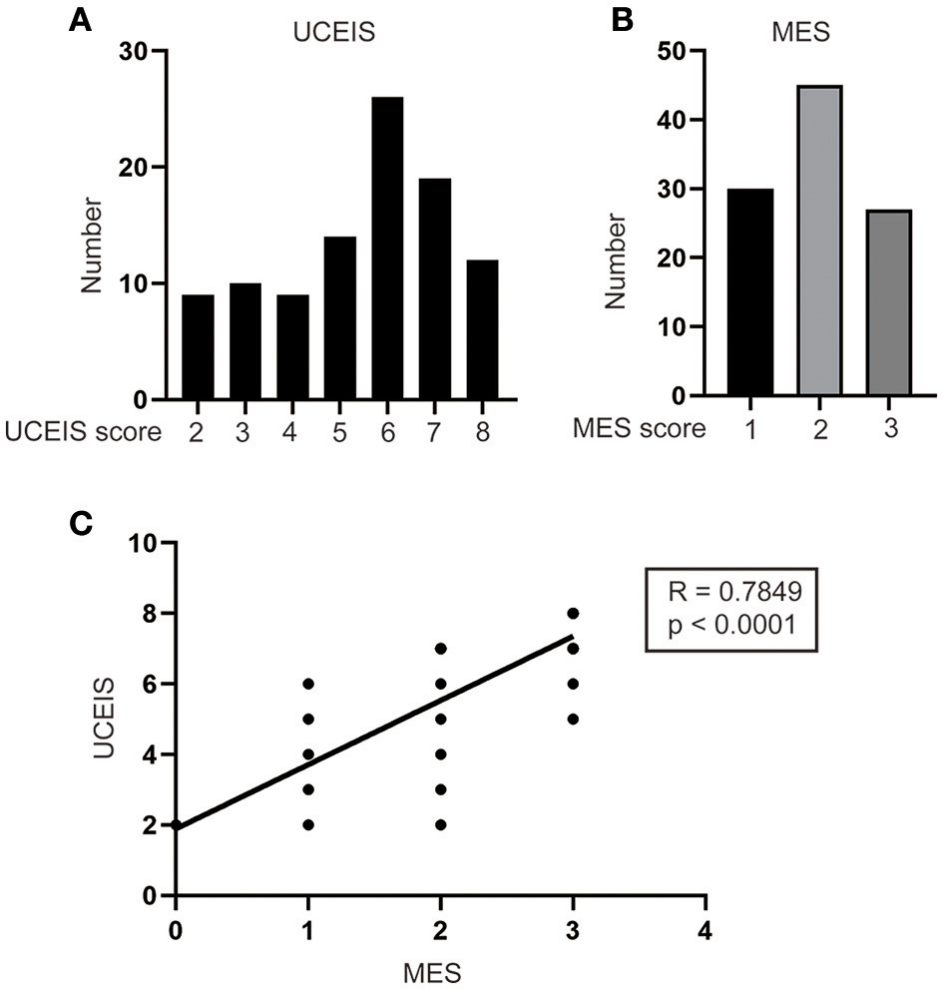
Analysis of the distribution and correlation of ulcerative colitis endoscopic index of severity (UCEIS) and Mayo endoscopic score (MES). The number of patients with different **(A)** UCEIS and **(B)** MES were analyzed. **(C)** Significant correlations existed between the UCEIS and MES (*R* = 0.7849, *p* < 0.0001).

A total of 566 patients with UC were treated at our institute. Among these patients, 450 patients underwent medicine treatment and 14 patients had IPAA without complete follow-up or clinical data. Thus, 102 (18.2%) patients with UC who received IPAA intervene were ultimately enrolled in this study. The flow diagram is shown in Figure 1. The median age at diagnosis was 40.0 years [interquartile range (IQR): 28.8–52.0) years with a median follow-up time of 5.0 (IQR: 2.0–9.0) years]. Among the patients, 1 (1.0%) patient was diagnosed as proctitis, 15 (14.7%) experienced left-sided colitis, and 86 (84.3%) developed pancolitis. In addition, 10 (9.8%) patients developed extraintestinal manifestation (EIM) during the disease course. The median UCEIS and MES of the patients were 6.0 (IQR: 4.0–7.0) and 2.0 (IQR: 1.0–3.0), respectively (Table 1). We further analyzed the number of patients with differing UCEIS and MES (Figures 2A,B) and the relationship between UCEIS and MES. As shown in Figure 2C, the association of UCEIS with MES was significant (*R* = 0.7849, *p* < 0.0001).

**Table 1:** Main baseline characteristics of patients.

**Variables**	**All cases (*n* = 102)**
Sex (male/female)	48/54
Age at diagnosis [yr, median (IQR)]	40.0 (28.8–52.0)
Disease duration [yr, median (IQR)]	4.0 (2.0–8.0)
Follow-up time [yr, median (IQR)]	5.0 (2.0–9.0)
Body mass index (kg/m2, mean ± SD)	19.6 ± 3.96
UCEIS [score, median (IQR)]	6.0 (4.0–7.0)
MES [score, median (IQR)]	2.0 (1.0–3.0)
Stage of surgery, *n* (%)	
II-stage IPAA	77 (75.5)
III-stage IPAA	25 (24.5)
Surgical approach, n (%)	
Open	58 (56.9)
Laparoscopic	44 (43.1)
Surgical urgency, n (%)	
Urgent surgery	9 (8.8)
Elective surgery	93 (91.2)
The reasons for IPAA	
Medicine failure	79 (77.5)
Medication intolerance	4 (3.9)
Serious complications	10 (9.8)
Malignant transformation	6 (5.9)
High drug-related expenses	4 (3.9)
Extraintestinal manifestations (EIMs), n (%)	10 (9.8)
History of surgery, n (%)	21 (20.6)
Extent of UC, n (%)	
E1	1 (1.0)
E2	15 (14.7)
E3	86 (84.3)
Mesalamine, n (%)	63 (61.8)
Biologics, n (%)	6 (5.9)
Steroids, n (%)	54 (52.9)
Immunomodulators, n (%)	17 (16.7)
Hb (g/L, mean ± SD)	104.7 ± 22.3
Alb (g/L, mean ± SD)	36.8 ± 6.1
Blood loss (ml, mean ± SD)	236.6 ± 196.3
Urine volume (ml, mean ± SD)	546.8 ± 415.2
Transfusion volume (ml, mean ± SD)	1,923.5 ± 771.8
Colloidal amount	841.2 ± 509.0

*UCEIS, Ulcerative colitis endoscopic index of severity; MES, Mayo endoscopic score; IPAA, ileal pouch-anal anastomosis; UC, ulcerative colitis; SD, standard deviation; Hb, hemoglobin; Alb, albumin*.

### Analysis of the Postoperative Complications of IPAA

A total of 34 (33.3%) patients presented with late postoperative complications. Of these patients, 22 (21.6%) developed pouchitis, 3 (2.9%) developed pouch failure, 7 (6.9%) experienced postoperative long-term intestinal obstruction, 4 (3.9%) had pouch–vagina leak, 4 (3.9%) developed anastomotic stricture, 1 (1.0%) had sexual dysfunction and 1 (1.0%) was diagnosed with infertility (Table 2). This demonstrated that pouchitis was the most common late complication of IPAA in this study.

**Table 2:** Main postoperative complications of IPAA.

**Complications**	**Number (%)**
**Early postoperative complications**	
Early postoperative Intestinal obstruction	8 (7.8)
Pouch and anastomotic bleeding	10 (9.8)
Pouch-anal anastomotic leak	3 (2.9)
Wound infection	11 (10.8)
Incision hernia	2 (2.0)
**Late postoperative complications**	
Pouchitis	22 (21.6)
Pouch failure	3 (2.9)
Postoperative long term intestinal obstruction	7 (6.9)
Pouch-vagina leak	4 (3.9)
Anastomotic stricture	4 (3.9)
Sexual dysfunction	1 (1.0)
Infertility	1 (1.0)

### Analysis of the Relationship Among UCEIS, MES, and PDAI

**Figure 3:**
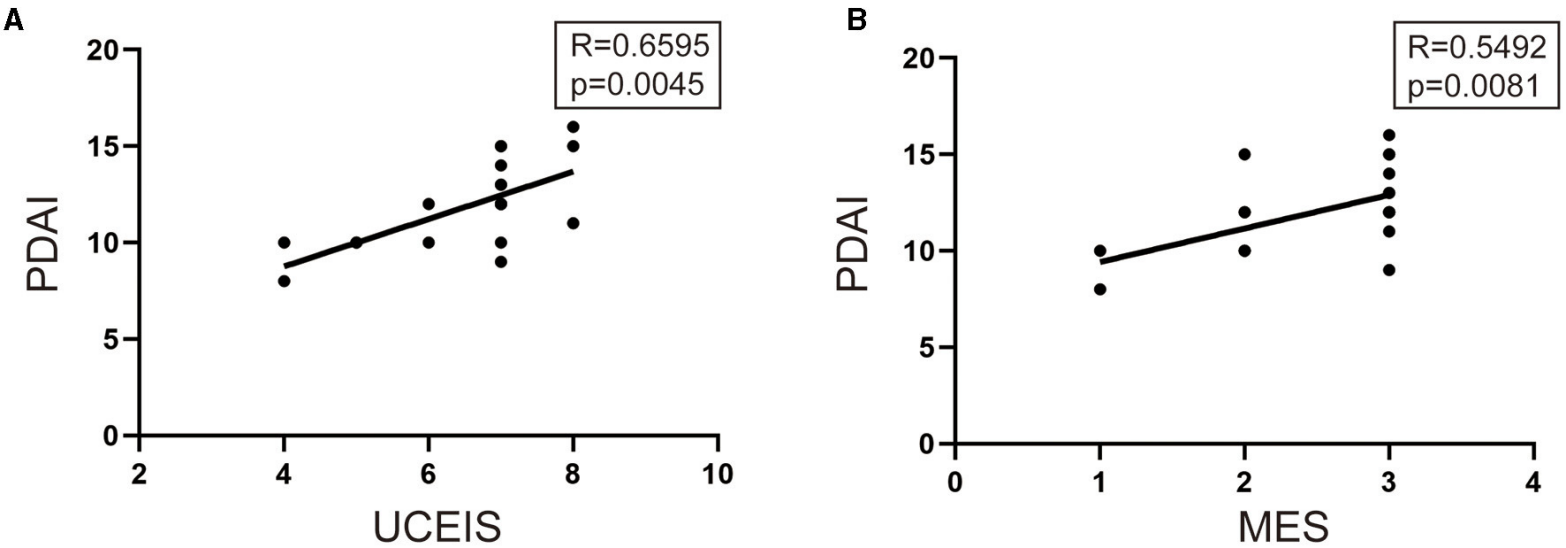
Pearson's correlation test was performed to analyze the relationship among UCEIS, MES, and pouchitis disease activity index (PDAI). **(A)** Significant correlations existed between the UCEIS and PDIA (*R* = 0.6595, *p* = 0.0045) and between **(B)** MES and PDAI (*R* = 0.5492, *p* = 0.0081).

To determine whether UCEIS or MES was associated with the PDAI, Pearson's correlation test was performed. As shown in Figures 3A,B, UCEIS demonstrated a stronger correlation with PDAI than MES (UCEIS: *R* = 0.6595, *p* = 0.0045 vs. MES: *R* = 0.5492, *p* = 0.0081).

### UCEIS and MES Threshold Evaluation for Predicting Pouchitis After IPAA

**Figure 4:**
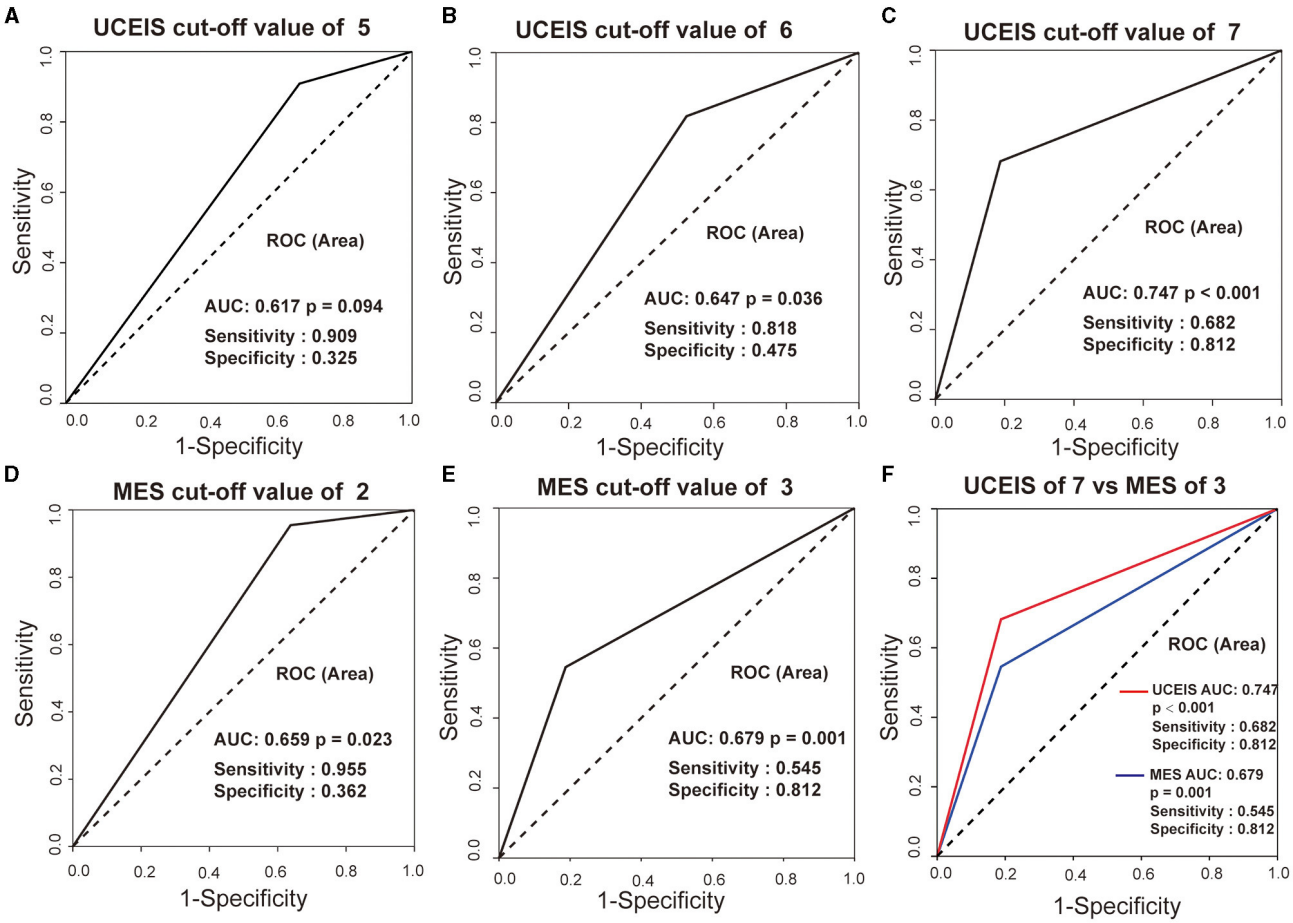
Receiver-operating characteristic (ROC) curves of UCEIS and MES in predicting pouchitis in patients with ulcerative colitis (UC). UCEIS had the most significant AUC of 0.747 with a sensitivity of 68.2%, and a specificity of 81.2% with a cut-off value of 7 **(A–C, F)**, while MES = 3 had an AUC of 0.679 with a sensitivity of 54.5% and specificity of 81.2% **(D, E, F)**.

To establish the best threshold value of UCEIS and MES for the indication of pouchitis, the ROC curve analysis was performed. As shown in Figure 4, UCEIS had the most significant area under the ROC curve (AUC) of 0.747 with a sensitivity of 68.2% and specificity of 81.2% at the cut-off value of 7 (*p* < 0.001), while MES of three had the biggest AUC of 0.679 with sensitivity of 54.5% and specificity 81.2% (*p* = 0.001). This indicates that UCEIS has a better predictive value for pouchitis than MES.

### Analysis of Post-IPAA Complications in Patients With Different MES and UCEIS

Based on the results of the ROC analysis, we have chosen UCEIS of seven and MES of three as cut-off values for further analysis of postoperative complications. We found that patients with MES = 3 were more likely to develop pouchitis than those with MES <3 (*p* = 0.002). We further found that patients with UCEIS ≥ 7 had a higher likelihood of developing postoperative pouchitis and long-term intestinal obstruction compared with those having UCEIS <7 (*p* < 0.001; *p* = 0.022) (Table 3).

**Table 3:** Analysis post-IPAA complications in differernt MES and UCEIS scores.

**Complications, *n* (%)**	**MES**		***p* value**	**UCEIS**		***p* value**
	**MES <3**	**MES of 3**		**UCEIS <7**	**UCEIS ≥ 7**	
**Early postoperative complications**						
Early postoperative Intestinal obstruction, *n* (%)			1.000^a^	67 (71.3)	27 (28/7)	0.690^a^
No	69 (73.4)	25 (26.6)		5 (62.5)	3 (37.5)	
Yes	6 (75.0)	2 (25.0)				
Pouch and anastomotic bleeding, *n* (%)			0.449^a^	65 (70.7)	27 (29.3)	1.000^a^
No	69 (75.0)	23 (25.0)		7 (70.0)	3 (30.0)	
Yes	6 (60.0)	4 (40.0)				
Wound infection			0.227^a^	65 (71.4)	26 (28.6)	0.727^a^
No	68 (74.7)	23 (25.3)		7 (63.6)	4 (36.4)	
Yes	7 (63.6)	4 (36.4)				
Pouch-anal anastomotic leak and incisional hernia, *n* (%)		0.606^a^	69 (71.1)	28 (28.9)	0.629^a^	
No	72 (74.2)	25 (25.8)		3 (60.0)	2 (40.0)	
Yes	3 (60.0)	2 (40.0)				
**Late complications**						
Pouchitis			0.002^b^	65 (81.3)	15 (18.7)	<0.001^b^
No	65 (81.3)	15 (18.7)		7 (31.8)	15 (68.2)	
Yes	10 (45.5)	12 (54.5)				
Postoperative long term intestinal obstruction, n (%)			0.078^a^	70 (73.7)	25 (26.3)	0.022^a^
No	72 (75.8)	23 (24.2)		2 (28.6)	5 (71.4)	
Yes	3 (42.9)	4 (57.1)				
Anastomotic stricture, n (%)			0.056^a^	70 (71.4)	28 (28.6)	0.579^a^
No	74 (75.5)	24 (24.5)		2 (50.0)	2 (50.0)	
Yes	1 (25.0)	3 (75.0)				
Pouch-vagina leak, n (%)			0.285^a^			0.579^a^
No	73 (74.5)	25 (25.5)		70 (71.4)	28 (28.6)	
Yes	2 (50.0)	2 (50.0)		2 (50.0)	2 (50.0)	

a *, Fisher's exact test; b, Chi-squared. IPAA, ileal pouch-anal anastomosis; MES, Mayo endoscopic score; UCEIS, Ulcerative colitis endoscopic index of severity*.

### UCEIS ≥ 7 and MES = 3 Were Independent Risk Factors for Pouchitis

Furthermore, we explored whether UCEIS ≥ 7 and MES = 3 were contributing factors to pouchitis. Univariate analysis revealed that UCEIS and MES were significantly associated with pouchitis (*p* < 0.001, *p* = 0.002) (Table 4). Multivariate logistic regression further demonstrated that UCEIS ≥ 7 and MES = 3 were both independent risk factors for pouchitis and patients with UCEIS ≥ 7 [odds ratio (OR), 8.860; 95% CI, 1.969–39.865, *p* < 0.001] had a higher risk of developing pouchitis than those with MES of 3 (OR, 5.200; 95% CI, 1.895–14.273; *p* = 0.001) (Table 4).

**Table 4:** Univariable and multivariate analysis of risk factors for pouchitis after IPAA in UC.

**Variables**	**Non-pouchitis group**	**Pouchitis group**	**Univariable analysis**	**Multivariate logistic regression**		
			***p* value**	**Odds Ratio**	**95% CI**	***p* value**
Sex, *n* (%)			0.812^a^			
Male	37 (77.1)	11 (22.9)				
Female	43 (79.6)	11 (20.4)				
Age at diagnosis, *n* (%)			0.336^a^			
<40y	36 (73.5)	13 (26.5)				
≥40y	44 (83.1)	9 (16.9)				
Disease duration, *n* (%)			0.811^a^			
<5y	44 (77.2)	13 (22.8)				
≥5y	36 (80.0)	9 (20.0)				
UCEIS, *n* (%)			<0.001^a^			
UCEIS <7	65 (90.3)	7 (9.7)				
UCEIS ≥7	15 (50.0)	15 (50.0)		8.860	1.969–39.865	<0.001
MES, *n* (%)			0.002^a^			
MES <3	65 (86.7)	10 (13.3)				
MES = 3	15 (55.6)	12 (44.4)		5.200	1.895–14.273	0.001
Conditions of relapse, *n* (%)			0.213^b^			
First occurrence	9 (100.0)	0 (0.0)				
First recurrence	39 (78.0)	11 (22.0)				
Multiple recurrence	32 (74.4)	11 (25.6)				
EIM, *n* (%)			0.446^c^			
No	73 (79.3)	19 (20.7)				
Yes	7 (70.0)	3 (30.0)				
Extent of UC, *n* (%)			0.752^b^			
E1	1 (100.0)	0 (0.0)				
E2	12 (80.0)	3 (20.0)				
E3	67 (77.9)	19 (22.1)				
History of surgery, *n* (%)			1.000^c^			
No	63 (77.8)	18 (22.2)				
Yes	17 (81.0)	4 (19.0)				
Hb, n (%)			0.464^a^			
≥ 110 g /L	31 (73.8)	11 (26.2)				
<110 g / L	49 (81.7)	11 (18.3)				
Alb, n (%)			1.000^c^			
≥ 35 g / L	54 (78.3)	15 (21.7)				
<35 g / L	26 (78.8)	7 (21.2)				
Hospitalization time, *n* (%)			0.229^c^			
<15 d	18 (90.0)	2 (10.0)				
≥ 15 d	62 (75.6)	20 (24.4)				
Stage of IPAA, *n* (%)			0.735^a^			
II-stage IPAA	61 (79.2)	16 (20.8)				
III-stage IPAA	19 (76.0)	6 (24.0)				
Blood loss, *n* (%)			1.000^a^			
<100ml	14 (77.8)	4 (22.2)				
≥ 100 ml	66 (78.6)	18 (21.4)				

a *, Chi-squared*;

b *, Wilcoxon's rank-sum test*;

c *, Fisher's exact test. IPAA, ileal pouch-anal anastomosis; UC, ulcerative colitis; MES, Mayo endoscopic score; UCEIS, Ulcerative colitis endoscopic index of severity; Hb, hemoglobin; Alb, albumin; EIM, extraintestinal manifestation*.

**Figure 5:**
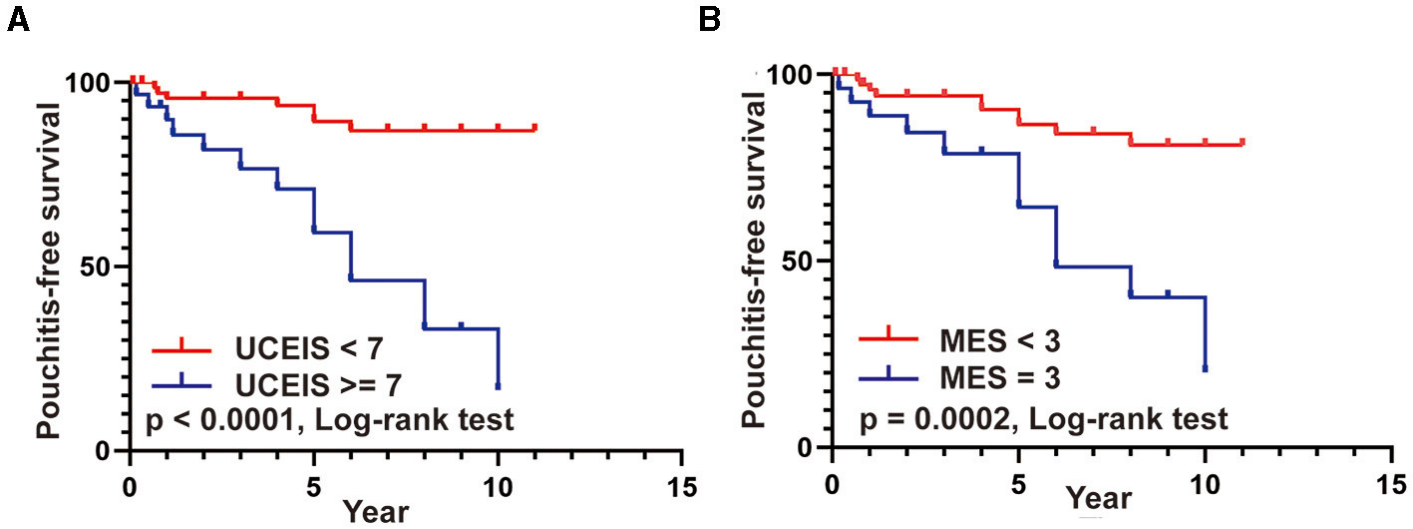
**(A,B)** Pouchitis-free survival rates in patients with UCEIS ≥ 7 vs. UCEIS <7 and MES = 3 vs. MES <3.

Moreover, we used the Kaplan–Meier method with the log-rank test to further compare the pouchitis-free survival rate in patients with different UCEIS and MES. As presented in Figures 5A,B, patients with UCEIS ≥ 7 and MES = 3 had significantly lower overall pouchitis-free survival than those with UCEIS <7 (*p* < 0.0001) and MES <3 (*p* = 0.002), respectively.

## Discussion

The UCEIS and MES were widely used systems to assess the endoscopic severity of UC to allow for convenient and appropriate decision-making by clinicians. However, whether UCEIS and MES can predict the development of pouchitis after IPAA and the comparison between their predictive values are still unclear. First, our study indicated that pouchitis occurred in 21.6% of IPAA cases in our institute and then demonstrated the predictive value of preoperative endoscopic activity on the occurrence of pouchitis. Furthermore, we proved that pre-IPAA UCEIS ≥ 7 and MES = 3 were contributing factors to pouchitis and that UCEIS outperformed MES as a predictor of pouchitis. These findings indicated the need for earlier and more frequent colonoscopic surveillance of the pouch after IPAA when patients had preoperative UCEIS ≥ 7.

Many previous studies have reported the risk factors for pouchitis (16, 17). The most recent study reported that patients with longer disease duration were susceptible to development of pouchitis (18). Similarly, researchers found that the cumulative incidence rates of pouchitis at 1, 2, 3, 4, and 5 years after IPAA were 25, 32, 36, 40, and 45%, respectively (19). Moreover, researchers reported that patients with cholangitis are more likely to undergo pouchitis surgery, with an incidence rate of 79% in the 10th year after IPAA (20). In addition, pancolitis, EIM, use of non-steroidal anti-inflammatory drugs, and specific gut microbiota were contributing factors to pouchitis (21–23). However, whether UCEIS and MES were associated with pouchitis was not reported in the literature. To the best of our knowledge, this study is the first to demonstrate that patients with UCEIS ≥ 7 had a higher risk of developing pouchitis than those with MES = 3. Previous study reported that histological and asymptomatic pouchitis had higher preoperative white blood cell count (*p* < 0.001) (24). Kalkana et al. discovered the relationship between the preoperative severity and activity of the disease and subsequent pouchitis development. They found that a higher disease activity index (*p* = 0.02) was an independent risk factors for the development of pouchitis (25). Therefore, we first constructed the endoscopic prediction model of preoperative UCEIS ≥ 7, which could reflect degree of the preoperative inflammation to provide a more direct and convenient approach to predict post-IPAA pouchitis to some extent.

Exploring novel predictors for pouchitis to enable early intervention is important in enhancing the management of UC after IPAA. Several recent studies have focused on the predictive value of fecal calprotectin (FC) for pouchitis. Johnson et al. indicated that the concentration of FC in pouchitis was significantly higher than that in uninflamed pouches and was associated with PDAI. Moreover, an FC cut-off value of 92.5 μg/g demonstrated a satisfactory predictive value for pouchitis (26). In another prospective study in which the FC level had already been evaluated before the diagnosis of pouchitis, an FC cut-off value of 56 μg/g exhibited a sensitivity of 100% and a specificity of 84% in predicting pouchitis (27). Thus, FC appeared to be a non-invasive and effective predictor of post-IPAA (28). However, the optimal threshold value of FC in predicting pouchitis and the timing of retesting of FC after IPAA has not been unified. In addition, not all patients will be tested for FC after IPAA, but all patients who undergo IPAA surgery will be subjected to preoperative endoscopic assessment for UCEIS and MES. We herein first indicated that UCEIS ≥ 7 had a better predictive value than MES = 3. Taken together, UCEIS outperformed MES in predicting pouchitis. Preoperative evaluation of endoscopic severity to determine UCEIS score could help clinicians predict the occurrence of post-IPAA pouchitis and earlier and more frequent postoperative colonoscopic surveillance could be conducted to prevent the development of pouchitis.

Limitations of this study included the loss to follow-up, which is inevitable in a retrospective study. Our sample size was relatively small. Larger sample sizes are recommended for future research.

## Conclusion

In this study, we discovered the incidence of post-IPAA pouchitis in our institute and demonstrated that the UCEIS performed better than MES in predicting post-IPAA pouchitis. We found that pre-IPAA UCEIS ≥ 7 had a significant association with PDAI and was an effective predictor of pouchitis. We further demonstrated that pre-IPAA UCEIS ≥ 7 was an independent risk factor for pouchitis with a higher risk than an MES = 3. Therefore, earlier and more frequent postoperative colonoscopic surveillance should be performed in patients with preoperative UCEIS ≥ 7 to prevent the occurrence of pouchitis and enhance its management.

## Data Availability Statement

The raw data supporting the conclusions of this article will be made available by the authors, without undue reservation.

## Ethics Statement

The studies involving human participants were reviewed and approved by The Ethics Committee of Xinhua Hospital (Approval No. XHEC-D-2020-107). The patients/participants provided their written informed consent to participate in this study.

## Author Contributions

PD and ZD conceived the study. WX analyzed the data and wrote the manuscript. WT and WD assisted some analysis. HH, WC, QQ, and LC collaborated to collect the information of patients. All authors participated in revising the manuscript and approved the final version.

## Funding

This work was supported by the National Natural Science Foundation of China (No. 82000481 and 81873547), the Shanghai Sailing Program (No. 20YF1429400), and the Supporting Project of the Medical Science and Technology Innovation Platform of Health Commission of Hubei Province (PTXM2020011).

## Conflict of Interest

The authors declare that the research was conducted in the absence of any commercial or financial relationships that could be construed as a potential conflict of interest.

## Publisher's Note

All claims expressed in this article are solely those of the authors and do not necessarily represent those of their affiliated organizations, or those of the publisher, the editors and the reviewers. Any product that may be evaluated in this article, or claim that may be made by its manufacturer, is not guaranteed or endorsed by the publisher.
